# Anaphylactic Shock After Intramuscular Diclofenac Sodium: A Case Report

**DOI:** 10.7759/cureus.55279

**Published:** 2024-02-29

**Authors:** Debashis P Sahoo, Bhupen Barman, Gwenette A War, Annu Gupta, Ruth K Tara

**Affiliations:** 1 Internal Medicine, All India Institute of Medical Sciences, Guwahati, Guwahati, IND; 2 Internal Medicine, North Eastern Indira Gandhi Regional Institute of Health and Medical Sciences, Shillong, IND; 3 General Medicine, All India Institute of Medical Sciences, Guwahati, Guwahati, IND; 4 General Medicine, North Eastern Indira Gandhi Regional Institute of Health and Medical Sciences, Shillong, IND

**Keywords:** adrenalin, in-hospital cpr, allergy and anaphylaxis, intramuscular drug use, diclofenac sodium

## Abstract

Diclofenac sodium is a commonly used nonsteroidal anti-inflammatory drug. It is widely used for acute and chronic pain management. Side effects, such as fixed drug eruption, asthmatic attack, and vasospastic angina, are commonly seen after the use of diclofenac sodium. However, anaphylaxis and anaphylactic shock secondary to injection of diclofenac sodium are rare. Our main aim in reporting this adverse event is to alert healthcare professionals to this potentially life-threatening adverse effect of diclofenac sodium and prompt use of adrenaline for treatment.

## Introduction

Diclofenac sodium is a commonly used analgesic for acute and chronic pain. It is a widely used non-steroidal anti-inflammatory drug (NSAID) for its antipyretic, anti-inflammatory, and analgesic properties [1]. It works by inhibiting cyclooxygenase-1 (COX-1) and cyclooxygenase-2 (COX-2). However, diclofenac sodium inhibits COX-2 more selectively, increasing the risk of gastrointestinal and cardiovascular events. All NSAIDs, especially COX-2 inhibitors, are associated with an increased risk of myocardial infarction, heart failure, ischemic stroke, Kounis syndrome, and death [2]. However, anaphylactic shock following diclofenac injection is rare [3]. This case report describes a life-threatening anaphylactic shock caused by intramuscular diclofenac sodium injection necessitating cardiopulmonary resuscitation (CPR) and adrenaline infusion.

## Case presentation

A 34-year-old man with a history of hypertension, hypothyroidism, and cervical spondylosis presented to the orthopedic outpatient department with severe neck pain that had been present for one month and had progressively worsened over the previous few days. He has no known allergies to drugs or any substances. He was given an intramuscular injection of diclofenac sodium 50 mg for acute pain relief while waiting for a cervical spine X-ray. A few minutes after the injection, the patient had breathing difficulties and subsequently lost consciousness. On examination, the patient was unresponsive to command and painful stimulus, with a feeble pulse and unrecordable blood pressure, was cyanosed, and his oxygen saturation was 30% in room air. He had widespread maculopapular rashes on his back and abdomen. Cardiac rhythm at the time of presentation showed ventricular fibrillation, for which immediate cardiopulmonary resuscitation (CPR) was initiated.

Immediate chest compressions with humidified oxygen were initiated by Ambu bag. Immediate defibrillation (200 J) was done and chest compressions were reinitiated. Intravenous injection of adrenalin 1 mg was given. The patient regained a weak pulse and blood pressure of 40/20 mm of Hg after being resuscitated with CPR. Endotracheal intubation was done and he was administered intramuscular adrenaline and intravenous hydrocortisone. The patient was then given an infusion of adrenaline and pheniramine maleate. He was shifted to the intensive care unit (ICU) and monitored. He regained full consciousness and achieved hemodynamic stability over the next few hours (blood pressure: 110/76 mmHg, pulse rate: 98/min with normal volume and character). The infusions were gradually tapered and stopped. He was shifted to the general ward after two days of ICU stay. After one day, he was discharged. 

## Discussion

Diclofenac is a drug approved by the United States Food and Drug Administration (FDA) that is commonly used to treat acute and chronic pain associated with musculoskeletal inflammatory conditions, such as rheumatoid arthritis, ankylosing spondylitis, and osteoarthritis. Its off-label uses include the treatment of migraine, myalgia, renal colic, gout, biliary colic, and postoperative pain. It belongs to the phenylacetic acid family and works to reduce inflammation. It acts by inhibiting the synthesis of inflammatory mediators of nociceptive response, such as prostaglandin E2 (PGE2), prostacyclins, and thromboxane (especially TXB2). It inhibits both cyclooxygenases competitively, but it also has COX-2 selective inhibition properties [4].

COX-1 is constitutively active in the human body, contributing to platelet activity, renal tissue perfusion, and gastric mucosa protection. However, COX-2 is an inducible enzyme that is activated in the presence of inflammatory mediators with nociceptive properties. It primarily affects synovial fluid and capsules [5]. It exerts peripheral analgesic action by downregulating the availability of sensitized pain receptors, which is attributed to stimulating cGMP pathway activation. However, inhibiting COX at other sites depletes protective substances in the stomach, resulting in gastric irritation. Its side effects include stomach pain, nausea, vomiting, melena, headache, drowsiness, and tinnitus [6]. It usually causes fixed drug eruptions, but it also causes urticaria, asthma attacks, and vasospastic angina [7]. Anaphylaxis and anaphylactic shock develop in some severe cases. It occurs primarily due to immune-mediated reactions ranging from delayed-type hypersensitivity and immunoglobulin E (IgE)-mediated anaphylaxis. It has previously been reported that this adverse event occurred following an intravenous injection of diclofenac. Our main aim in reporting this adverse event is to raise awareness among healthcare professionals about one of the most life-threatening adverse effects of diclofenac sodium, even when used intramuscularly and adrenaline should be used promptly to treat anaphylaxis.

## Conclusions

Diclofoenac sodium-induced hypersensitivity may range from simple urticaria to very severe anaphylactic shock. These reactions are more common in individuals with a history of allergies and having autoimmune diseases. As a result, even before administering a common analgesic, a thorough allergic history should be obtained, and the patient should be monitored for some time after the analgesic has been administered.
